# A Huge Cystic Retroperitoneal Lymphangioma Presenting with Back Pain

**DOI:** 10.1155/2016/1618393

**Published:** 2016-10-23

**Authors:** Mahir Gachabayov, Kubach Kubachev, Elbrus Abdullaev, Valentin Babyshin, Dmitriy Neronov, Abakar Abdullaev

**Affiliations:** Department of Abdominal Surgery, Vladimir City Clinical Hospital of Emergency Medicine, Gorky Street 5, Vladimir 600017, Russia

## Abstract

Retroperitoneal lymphangioma is a rare location and type of benign abdominal tumors. The clinical presentation of this rare disease is nonspecific, ranging from abdominal distention to sepsis. Here we present a 73-year-old female patient with 3-month history of back pain. USG and CT revealed a huge cystic mass which was surgically excised and appeared to be lymphangioma on histopathology.

## 1. Introduction

Lymphangioma is a benign neoplasm formed as a result of congenital malformation of lymphatic vessels leading to lymphangiectasia [1]. The exact incidence of retroperitoneal lymphangiomas is unknown due to its rarity. They are more common in children and more frequent in boys (M : F ratio is 5 : 2) [2, 3]. Among all patients, pancreatic lymphangiomas were shown to be encountered more commonly in female patients with the female-to-male ratio of 29 : 16 and with the mean age of 40 years [4]. 75% of lymphangiomas are encountered in the neck and 20% in the axillary region, and only 5% are intra-abdominal including spleen, liver, pancreas, and very rarely in the retroperitoneum [5]. Retroperitoneal location being one of the rarest locations accounts for about 1% of all lymphangiomas [6]. Clinical presentation of retroperitoneal lymphangioma is diverse, from asymptomatic to bowel obstruction, sepsis, and disseminated intravascular coagulopathy [7, 8]. Back pain is also a rare clinical presentation of this rare clinical entity.

## 2. Case Presentation

**Figure 1:**
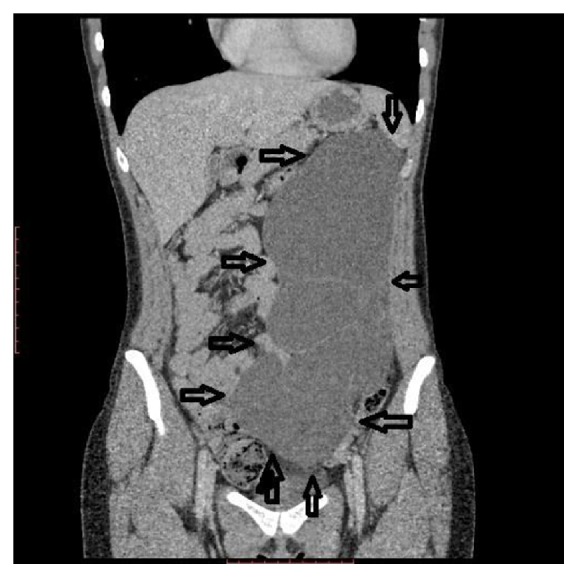
CT abdomen, coronal plane showing huge cystic retroperitoneal lymphangioma.

**Figure 2:**
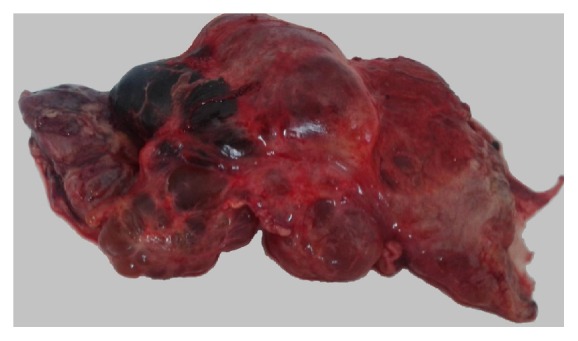
Lymphangioma, postexcision.

**Figure 3:**
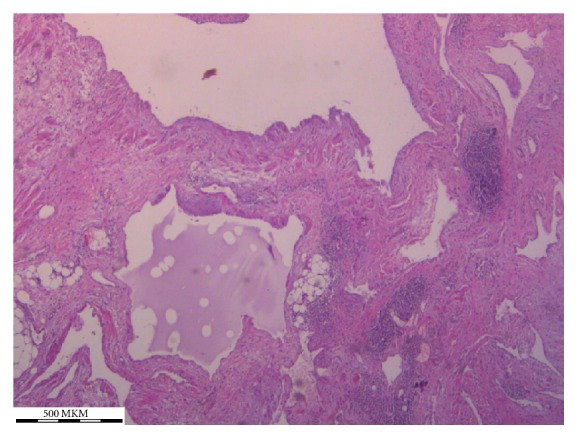
Photomicrograph of lymphangioma walls lined by endothelial cells containing aggregates of lymphocytes.

A 73-year-old female patient was examined for left-sided back pain which she had noticed during the last 2-3 months. Concomitant diseases the patient had included controlled hypertension and type 2 diabetes mellitus. Laboratory findings were insignificant. During abdominal examination a huge palpable nontender mass occupying the left half of the abdomen was found. Abdominal ultrasound was performed which showed a massive multilocular cyst (see Video 1 of the Supplementary Material available online at http://dx.doi.org/10.1155/2016/1618393). Abdominal CT was performed to identify the exact location and the relationship to adjacent organs which revealed a huge retroperitoneal septate multilocular cystic lesion with the maximal diameter of 31 cm occupying almost the entire left half of the abdominal cavity (Figure 1, Video 2, Video 3). The patient was operated on; laparotomy and excision of the cystic lesion, which had no connection with retroperitoneal organs, were performed (Figure 2). The histopathological exam showed the cystic lesion to be a lymphangioma (Figure 3). The postoperative course was uneventful and the patient was discharged on the 10th postoperative day without back pain. On the follow-up after 5 months the patient was good; no recurrence was found sonographically.

## 3. Discussion

Lymphangioma was first described by Koch in 1913 [9, 10]. Regarding the etiology of lymphangiomas a well-established theory suggests that they develop from a congenital malformation of lymphatic vessels, leading to blockage of lymphatic flow and lymphangiectasia [11, 12]. However, other possible causes, especially in adults, have been suggested to be inflammation, surgery, radiotherapy, and abdominal trauma [13–15]. Three histological types of lymphangiomas are present: cystic, capillary, and cavernous [16]. Retroperitoneal lymphangioma is commonly of cystic type [17].

Clinical presentation of retroperitoneal or abdominal lymphangiomas is variable with no pathognomonic signs and symptoms [18]. Generally they are asymptomatic with the first symptoms being abdominal distention, mild abdominal pain, abdominal asymmetry due to enlarging mass. In some cases they are revealed incidentally during abdominal examination or radiology for another abdominal condition. However, there are several reported cases of lymphangiomas leading to surgical emergencies, such as hemorrhage, bowel obstruction, ureteric obstruction, and sepsis [7, 8, 19, 20]. Lesser sac lymphangioma was reported to mimic acute appendicitis [21]. Such rare clinical manifestations as anemia and back pain have also been reported [22–24]. Our patient first noticed left-sided back pain during 2-3 months which emerged probably due to enlarging mass compressing retroperitoneal tissues.

Differential diagnoses of cystic retroperitoneal lymphangioma include retroperitoneal hematoma, abscess, duplication cysts, ovarian cysts, microcystic pancreatic adenoma, pancreatic pseudocysts, mucinous pancreatic neoplasms, branch-type IPMN, lymphangiosarcoma, cystic metastases (especially from ovarian and gastric primaries), undifferentiated sarcoma, cystic teratoma, cystic mesothelioma, and malignant mesenchymoma [25–27].

Abdominal USG generally shows unilocular or multilocular anechoic masses, in some cases containing echoic debris of calcifications, with multiple thin septae presenting in honeycomb or cobweb pattern [28]. Contrast-enhanced CT may show cystic mass with enhancement of the wall and septa containing homogenous fluid with low attenuation values. Negative attenuation values can occur due to chylous content [29]. Calcification can occur but is uncommon [29, 30]. The MRI findings of lymphangioma are described as well-defined, round, or oval and density uniformity cystic masses and long signal intensity on T1- and T2-weighted images [18, 31]. Lymphoscintigraphy SPECT/CT has been shown to be an accurate radiologic exam [32]. However, this technology is expensive and not widely available. Several staging systems based on predicted prognosis and surgical results were also developed [33]. Despite the presence of several modalities of radiology, they all can only help to determine sizes, location, presence of invasion, and characteristics of contents. They are insufficient to establish an accurate preoperative diagnosis [15]. The definitive diagnosis of lymphangioma is histopathology and immunohistochemistry after its surgical excision. Double staining with Prox1 and CD31 is the most reliable method for characterizing lymphangioma endothelial cells [15, 34].

The treatment of retroperitoneal cystic lymphangioma is its surgical excision which can be performed via either laparotomy or laparoscopy. In case of complicated cyst or close relation with abdominal organs the extent of procedure could be extended, such as bowel resection [35]. Ishibashi et al. have recently shown the use of double balloon catheter to be useful in minimizing the risk of the spillage of cyst contents into the peritoneal or retroperitoneal cavity [36]. The nonoperative treatment by aspiration of contents and injection of sclerosant agents is another modality that has been demonstrated to be effective [37]. However, it is worth emphasizing that a 10% recurrence rate is the result of nonradical excision as evidenced by positive margins at histopathology [38]. Therefore total surgical excision is the treatment of choice to avoid recurrence, progressive growth, infection, rupture, and bleeding [38, 39].

To conclude, cystic retroperitoneal lymphangioma, especially when presented with back pain, is a rare clinical entity which surgeons should be aware of. The definitive treatment of lymphangiomas is total surgical excision.

## Supplementary Material

Video 1. Abdominal USG showing a huge multiseptate retroperitoneal cystic mass. Video 2. CT abdomen, axial plane showing a huge multiseptate retroperitoneal cystic mass. Video 3. CT abdomen, coronal plane showing a huge multiseptate retroperitoneal cystic mass.
